# A p53-independent role for the MDM2 antagonist Nutlin-3 in DNA damage response initiation

**DOI:** 10.1186/1471-2407-11-79

**Published:** 2011-02-21

**Authors:** Jane M Valentine, Sonia Kumar, Abdeladim Moumen

**Affiliations:** 1 DNA Damage Response Group, Basic Medical Science Department, St George's University of London, Cranmer Terrace, London, UK; 2 Outreach for Medicine, Department of Medical Education, School of Medicine, King's College London, 4.20 Shepherd's House, Guy's Campus, London Bridge, London, UK

## Abstract

**Background:**

The mammalian DNA-damage response (DDR) has evolved to protect genome stability and maximize cell survival following DNA-damage. One of the key regulators of the DDR is p53, itself tightly regulated by MDM2. Following double-strand DNA breaks (DSBs), mediators including ATM are recruited to the site of DNA-damage. Subsequent phosphorylation of p53 by ATM and ATM-induced CHK2 results in p53 stabilization, ultimately intensifying transcription of p53-responsive genes involved in DNA repair, cell-cycle checkpoint control and apoptosis.

**Methods:**

In the current study, we investigated the stabilization and activation of p53 and associated DDR proteins in response to treatment of human colorectal cancer cells (HCT116^p53+/+^) with the MDM2 antagonist, Nutlin-3.

**Results:**

Using immunoblotting, Nutlin-3 was observed to stabilize p53, and activate p53 target proteins. Unexpectedly, Nutlin-3 also mediated phosphorylation of p53 at key DNA-damage-specific serine residues (Ser15, 20 and 37). Furthermore, Nutlin-3 induced activation of CHK2 and ATM - proteins required for DNA-damage-dependent phosphorylation and activation of p53, and the phosphorylation of BRCA1 and H2AX - proteins known to be activated specifically in response to DNA damage. Indeed, using immunofluorescent labeling, Nutlin-3 was seen to induce formation of γH2AX foci, an early hallmark of the DDR. Moreover, Nutlin-3 induced phosphorylation of key DDR proteins, initiated cell cycle arrest and led to formation of γH2AX foci in cells lacking p53, whilst γH2AX foci were also noted in MDM2-deficient cells.

**Conclusion:**

To our knowledge, this is the first solid evidence showing a secondary role for Nutlin-3 as a DDR triggering agent, independent of p53 status, and unrelated to its role as an MDM2 antagonist.

## Background

The p53 tumour suppressor protein, often referred to as the '*guardian of the genom*', plays a critical role in mediating cellular stress responses such as that brought about by DNA-damage, and is therefore key in regulating a vast array of proteins involved in cell cycle progression and check-points, DNA repair and apoptosis [1].

**Figure 1:**
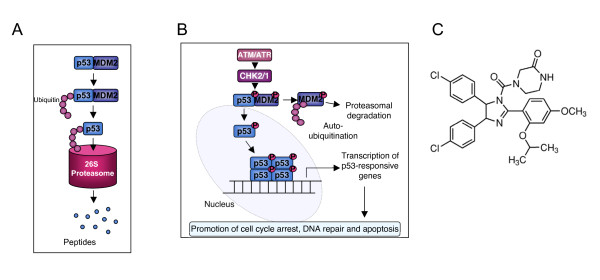
**Schematic representation of the interactions between p53 and MDM2**. *(A) *In the absence of stress signals, p53 is bound to its negative regulator MDM2. MDM2 ubiquitinates p53, targeting it for degradation by the 26 S proteasome. *(B) *Cellular stress signals, such as that bought about by DNA-damage lead to activation of ATM/ATR. ATM/ATR mediate the phosphorylation of MDM2 and p53. Phosphorylated MDM2 undergoes auto-ubiquitination and degradation by the 26 S proteasome. Phosphorylated p53 undergoes nuclear localisation, tetramerisation, and binds to p53-responsive promoters to induce transcription of genes involved in the DDR. *(C) *Chemical structure of Nutlin-3.

In the absence of cellular stress, p53 is maintained at low levels by its ubiquitination and subsequent proteasomal degradation. This process can be mediated by one of several E3 ubiquitin ligases [2], but principally by MDM2 (mouse double minute 2), as illustrated in Figure 1A.

Conversely, in the presence of cellular stress stimuli, two protein kinases - ATM (ataxia-telangiectasia mutated) and ATR (ATM and Rad3-related) orchestrate the DDR in order to preserve genome integrity. Whilst ATM is mainly activated in response to double-strand DNA breaks (DSBs), ATR is primarily activated following replicative errors that result in single-stranded DNA, however recent findings indicate DSB-mediated activation of ATM can also trigger activation of ATR [3,4].

Activation of ATM leads to phosphorylation and activation of CHK2, along with various other substrates, resulting in the subsequent phosphorylation of both p53 and its negative regulator MDM2 (Figure 1B). Phosphorylation of MDM2 in close proximity to its RING domain inhibits its ability to ubiquitinate p53, instead promoting self-ubiquitination and degradation by the proteasome.

Conversely, the phosphorylation of p53 results in its stabilisation and activation [5-7], bringing about its translocation to the nucleus, where it has been shown to bind preferentially to promoters which favour transcription of genes that encode proteins required in stress-induced cell cycle check-point control, DNA repair and apoptosis. Adding to the complexity of p53-mediated DDR signalling are several reports indicating that co-operation of p53 with other transcription factors such as hnRNP K and Miz-1 is necessary for the efficient transcription of some p53 target genes, particularly those encoding apoptogenic proteins [8-10].

The functional roles of p53 phosphorylation vary and are yet to be fully elucidated. Evidence suggests that phosphorylation of p53 at Ser20 leads to inhibition of the p53/MDM2 interaction, preventing ubiquitin-mediated p53 degradation and thereby enhancing p53 stabilisation [11-13]. On the other hand, phosphorylation of p53 at Ser46 has been shown to mediate the selectivity of p53 in favour of promoters which enhance apoptotic signalling, such as the p53-regulated apoptosis-inducing protein 1 (p53AIP) [14]. Furthermore, certain phosphorylations provide a means of negatively regulating p53, as evidenced by observations that phosphorylation of p53 at Thr55 inhibits its nuclear localisation [15] and mediates its degradation [16], whilst dephosphorylation of nuclear p53 at Ser276 has been observed to occur as an early response to ionising radiation [17].

There also exists much debate as to whether specific phosphorylations are prerequisite for the stabilisation and functional activity of p53. Findings in U2OS osteoblast cells show that isopropyl-ß-D-thiogalactoside-induced (IPTG) sequestration of MDM2 by p14/ARF led to phosphorylation of only a single p53 residue; Ser392, whilst adriamycin caused phosphorylation of all 6 key serine residues (Ser6, 10, 15, 20, 37 and 392), but no differences were observed between the activity of p53 in adriamycin versus IPTG-treated cells, seemingly indicating that phosphorylation is not necessary for p53 activity [18]. However, Chehab *et al *observed complete ablation of p53 stabilisation in response to UV treatment or irradiation in cells where Ser20 was substituted for alanine or aspartate [11].

Given the vast array of proteins under the regulation of p53, and the fact that mutations to p53 are present in over 50% of all human malignancies [19,20], there is much interest in developing pharmacological agents directed at p53-mediated responses. Recently, a novel small molecule MDM2 antagonist has been developed; Nutlin-3 (Figure 1C) interacts with the p53 binding domain of MDM2, preventing negative regulation of p53 by MDM2, hence allowing continuation of p53-mediated signalling [21]. Studies by the same group also showed that Nutlin-3 treatment of p53-positive HCT116 and RKO cells enhanced transcription of p53-responsive genes including p21, MIC1 and MDM2, leading to the initiation of apoptosis, despite the fact that no phosphorylation of p53 was observed at a number of key serine residues (Ser6, 15, 20, 37, 46 and 392) [22]. The authors attribute their findings to the proposed non-genotoxic action of Nutlin-3, however Nutlin-3-induced phosphorylation of p53 at Ser15 has since been reported in both B-cell chronic lymphocytic leukaemia (B-CLL) and mantle cell lymphoma (MCL) models [23].

In the current study we assessed the stabilisation and activation of p53 in HCT116^p53+/+ ^cells in response to Nutlin-3, finding significant phosphorylation of Ser15, along with Ser20 and Ser37. Furthermore, on investigation of other components of the DDR pathway, we show Nutlin-3-mediated activation of ATM, CHK2, BRCA1 and H2AX, as well as upregulation of MDM2 and p21. Nutlin-3 led to G1/S arrest in HCT116^p53+/+ ^cells, in keeping with the established role of p53 in instigating and maintaining G1 arrest, however in HCT116^p53-/- ^cells, G2/M arrest was noted in response to Nutlin-3 treatment, demonstrating the ability of Nutlin-3 to induce cell cycle checkpoint controls in a p53-independent fashion. Additionally, in response to Nutlin-3, we show nuclear H2AX foci formation, an early event in the DDR caused by clustering of phosphorylated H2AX moieties (γH2AX) at the site of DSBs. Moreover, this phenomenon was also observed in HCT116 cells lacking p53 (HCT116^p53-/-^) and also in MDM2 deficient cells (MEF^MDM2-/-^), suggesting firstly that p53 status is dispensable in the Nutlin-3-induced DDR, and secondly, that the ability of Nutlin-3 to induce DNA-damage or initiate the DDR is not connected to its role as an MDM2 antagonist. These results suggest a secondary role for Nutlin-3 as a DNA-damaging agent, contrary to its proposed mechanism of action as a non-genotoxic antagonist of MDM2. These data have implications for the use of Nutlin-3, and for the future development of pharmacological MDM2 antagonists for the treatment of cancer.

## Methods

Unless otherwise stated all antibodies were purchased from New England Biolabs, Hertfordshire, UK, and all reagents, including Nutlin-3, were purchased from Sigma-Aldrich, Dorset, UK.

### Cell Lines

Human colorectal cancer cell lines (HCT116^p53-/- ^and HCT116^p53+/+^) were obtained from Professor Galina Selivanova (Karolinska Institute, Stockholm, Sweden), and mouse embryonic fibroblast (MEF) cells deficient in MDM2 (MEF^MDM2-/-^) were obtained from Professor Guillermina Lozano (MD Anderson Cancer Centre, University of Texas, USA). All cells were genotyped before arrival using where necessary primers specific to the deleted alleles. Cell lines were authenticated upon receipt using immunoblotting. Cells were sustained in Dulbecco's Modified Eagle's Medium, supplemented with 10% fetal bovine serum, 1% Penicillin/Streptomycin/L-Glutamine and 1% Amphotericin B (Invitrogen, Renfrewshire, UK). Cells were incubated at 37°C in a humidified atmosphere containing 5% CO_2_. Cells were passaged twice weekly, and were seeded at 1 × 10^5^cells/mL during all experiments.

### Western Blotting

Following varying length treatments with 10 μM Nutlin-3 or 100 μM Etoposide, cells were collected and lysed in 2X laemmli lysis buffer (4% w/v SDS, 20% v/v glycerol, 120 mM tris pH6.8). A 5 μL volume of each sample was diluted in 95 μL dH_2_0, and added to 1 mL Lowry solution (50 parts 2% w/v sodium carbonate, 0.1 M sodium hydroxide solution, to 1 part 0.5% w/v copper(II)sulphate, 1% w/v sodium citrate solution), incubated at room temperature for 10 minutes, added to 100 μL 1 M folinciocalteau solution, and incubated for 30 minutes at room temperature before being transferred to cuvettes for determination of protein concentration using a CamSpec-M330 spectrometer. Samples of equal protein concentration were then loaded onto 6-15% acrylamide gels and underwent electrophoresis, followed by transfer onto PVDF (polyvinylidene fluoride) membranes. Membranes were blocked with 5% milk/TBS-T solution (5%w/v Marvel milk powder in 1X TBS-T solution comprising 50 mM Tris, 150 Mm sodium chloride, 0.364% v/v hydrochloric acid, 0.5% v/v Tween-20) and probed overnight at 4°C for specific proteins of interest. Standard primary antibody dilutions were 1:1000 in 5% milk/TBS-T solution, except for CHK2 (1:100 in 5% BSA/TBS-T solution), tubulin and actin (Merck Chemicals, Nottinghamshire, UK), used at 1:17000 in 5% milk/TBS-T solution. Standard secondary antibody dilutions were 1:2000 prepared in 5% milk/TBS-T solution. Chemiluminescence was detected using Lumiglo reagent (New England Biolabs, Hertfordshire, UK) according to manufacturer's instructions, and hyperfilms (GE Healthcare, Buckinghamshire, UK) were developed using an Amersham SRX100A Hyperprocessor.

### Flow Cytometry

After treatment with 100 μM Etoposide or 10 μM Nutlin-3 for various time periods, cells were trypsinised using 0.05% EDTA-free trypsin (Invitrogen, Renfrewshire, UK), collected and centrifuged, and the pellets resuspended in 70% ethanol before being stored for 24 hours at -20°C. Cells were later centrifuged, washed with 1X PBS and resuspended in 50 μg/mL Propidium Iodide/Rnase A solution before cell cycle distribution was assessed on a Beckman Coulter Cytomics FC500 flow cytometer.

### Immunofluorescence

Cells in 6-well plates were treated with 100 μM Etoposide or 10 μM Nutlin-3 for varying time periods before being fixed using 4% v/v paraformaldehyde solution, permeabilised with 0.5% v/v Triton-X100 solution, washed in 1X PBS and incubated overnight at 4°C with various antibodies prepared in 5% milk/TBS-T solution. Cells were then washed with 1X PBS, incubated for 2 hours with a 1:250 dilution of goat anti-rabbit Dylight488 antibody (New England Biolabs, Hertfordshire, UK) prepared in 1X PBS, before being washed once again with 1X PBS. Wells were then treated with one drop of Vectashield mounting media containing DAPI (Vector Laboratories, Cambridgeshire, UK), covered with glass coverslips and sealed with clear nail polish. Cells were then observed at 40× magnification using a Zeiss LSM500 confocal microscope and analysed using LSM Image Browser software (Carl Zeiss, Oberkochen, Germany).

## Results

### Nutlin-3 induces stabilisation of p53 and activation of p53 target proteins

**Figure 2:**
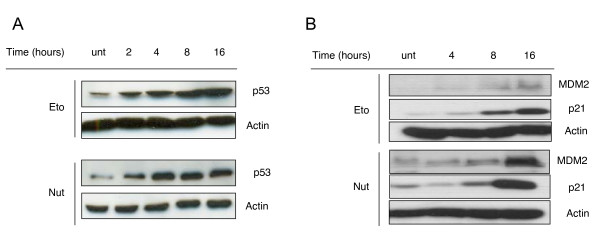
**Nutlin-3 induces stabilisation and phosphorylation of p53, and activates key p53 target proteins**. *(A) *HCT116^p53+/+ ^cells were untreated (treated with DMSO only) (unt) or treated with 100 μM Etoposide (Eto), or 10 μM Nutlin-3 (Nut) for the times indicated before immunoblotting was used to analyze p53 stabilisation. Actin levels were used to assess equal loading. *(B) *HCT116^p53+/+ ^cells were treated with 100 μM Etoposide (Eto) or 10 μM Nutlin-3 (Nut) for the times indicated before immunoblotting was used to analyse MDM2 and p21 activation. Actin levels were used to assess equal loading. *(C) *HCT116^p53-/- ^cells were untreated (treated with DMSO only) (unt) or treated 10 μM Nutlin-3 (Nut) for the times indicated before immunoblotting was used to analyze p53 stabilisation. Actin levels were used to assess equal loading.

In order to compare the efficiency of Nutlin-3-dependent p53 stabilisation with that of known DNA-damaging agents, we treated human colorectal cancer cells (HCT116^p53+/+^) with Etoposide (100 μM) or Nutlin-3 (10 μM). Treatment of HCT116^p53+/+ ^cells with these different agents led to stabilisation of p53 from 2 hours. Stabilisation of p53 was still apparent after 16 hours in cells treated with either Etoposide or Nutlin-3 (Figure 2A). As expected, no p53 was observed in HCT116^p53 -/- ^cells treated with any of the two reagents throughout the time course examined (Figure 2C).

Given that we observed stabilisation of p53 in response to Nutlin-3, we sought to identify whether Nutlin-3 caused activation of p53 target proteins; MDM2 and p21. Indeed, following 4, 8 or 16 hour treatments with Nutlin-3, activation of p21 was observed to be similar to that induced by Etoposide. Additionally, Nutlin-3-induced activation of MDM2 greatly exceeded that resulting from Etoposide treatment throughout the time course studied (Figure 2B).

### Nutlin-3 induces phosphorylation of p53 at key serine residues and activates several important DDR mediators

**Figure 3:**
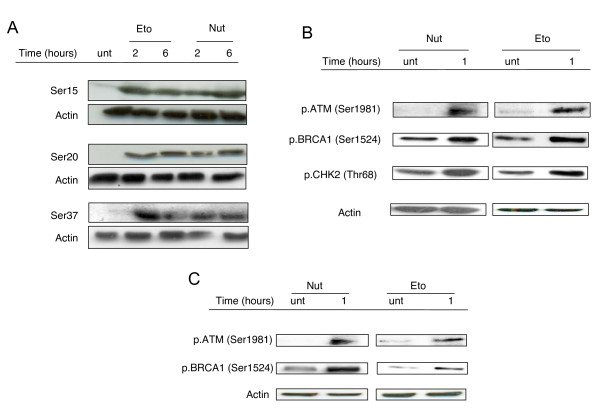
**Nutlin-3 leads to phosphorylation of several important DDR mediators**. *(A) *HCT116^p53+/+ ^cells were untreated (treated with DMSO only) (unt) or treated with 100 μM Etoposide (Eto) or 10 μM Nutlin-3 (Nut) for the times indicated before immunoblotting was used to analyse phosphorylation of p53 at Ser15, Ser20 and Ser37. Actin levels were used to assess equal loading. *(B) *HCT116^p53+/+ ^cells were untreated (treated with DMSO only) (unt) or treated with 10 μM Nutlin-3 (Nut) or 100 μM of Etoposide (Eto) for 1 hour before the phosphorylation of ATM (Ser1981), BRCA1 (Ser1542) and CHK2 (Thr68) were analysed using immunoblotting. Actin levels were used to assess equal loading. *(C) *HCT116^p53-/- ^cells were untreated (treated with DMSO only) (unt) or treated with 10 μM Nutlin-3 or 100 μM of Etoposide (Eto) for 1 hour before the phosphorylation of ATM (Ser1981) and BRCA1 (Ser1542) were analysed using immunoblotting. Actin levels were used to assess equal loading.

Following the observed stabilisation of p53 in HCT116^p53+/+ ^cells induced by treatment with Etoposide or Nutlin-3, we next sought to investigate whether or not the observed Nutlin-3-dependent stabilisation of p53 was a result of Nutlin-3-induced p53 phosphorylation. Therefore, the phosphorylation status of various key serine residues known to be phosphorylated following DNA-damage was examined in response to the same two reagents over a 24 hours time-course. Indeed, phosphorylation of Ser15, 20 and 37 was observed at both 2 and 6 hour time points in response to Etoposide and Nutlin-3 treatment (Figure 3A). However a marked decrease in p53 phosphorylation was observed following Nutlin-3 treatment at 24 hour point (Additional file 1).

Since it is well established that Etoposide-dependent phosphorylation of p53 is a response to DNA-damage generated by this agent, we went on to investigate whether the unexpected Nutlin-3-induced p53 phosphorylation was due to a Nutlin-3-mediated DDR. Therefore, we assessed the affect of Nutlin-3 on the activation of CHK2 and ATM which are required for DNA-damage-dependent phosphorylation and activation of p53. Indeed, phosphorylation of ATM and CHK2 were observed in HCT116^p53+/+ ^cells following 1 hour treatments with either Etoposide or Nutlin-3, as was phosphorylation of BRCA1, an ATM target protein required for the ATM-dependent DDR (Figure 3B). Furthermore, in HCT116^p53-/- ^cells, phosphorylation of both ATM and its target protein BRCA1 was also noted following a 1 hour treatment with both Nutlin-3 and Etoposide (Figure 3C).

### Nutlin-3 induces G1/S cell cycle arrest

**Figure 4:**
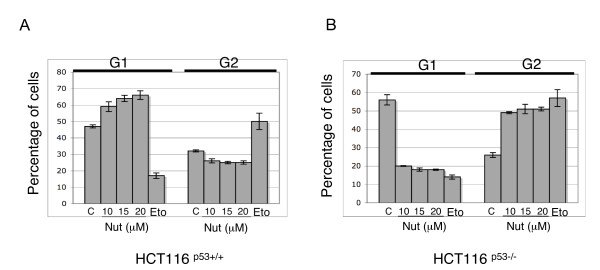
**Nutlin-3 induces p53-independent cell cycle checkpoint controls**. HCT116^p53+/+ ^and HCT116^p53-/- ^cells were treated with either 0, 10, 15 or 20 μM Nutlin-3 (Nut) or 100 μM of Etoposide (Eto). After 18 hours, cell cycle distribution was assessed using flow cytometry. *(A) *Chart showing percentage of HCT116^p53+/+ ^cells in either G1 or G2 cell cycle population following either Nutlin-3 or etoposide treatment as described above. *(B) *Chart showing percentage of HCT116^p53-/- ^cells in both G1 and G2 cell cycle population following either Nutlin-3 or Etoposide treatment as described above.

Given our findings that Nutlin-3 treatment induced p53 stabilisation and phosphorylation, as well as the activation of key DDR proteins and p53 target proteins known to be involved in cell cycle control, we went on to assess whether Nutlin-3 was capable of inducing cell cycle checkpoints. Following treatment with either Nutlin-3 or Etoptoside, HCT116^p53+/+ ^and^p53-/- ^cells were analysed by flow cytometry. While HCT116^p53+/+ ^treatment with Nutlin-3 led to G1/S arrest, treatment with Etoposide led to G2/M arrest (Figure 4A, Additional file 2 and Table 1). In contrast HCT116 p53-/- cells were observed to arrest in G2/M in response to both Nutlin-3 and Etoposide (Figure 4A, Additional file 2 and Table 2). Furthermore and in contrast to HCT116^p53+/+^, an increase in subG1 cell population was observed in HCT116^p53-/-^following Nutlin-3 treatment (Figure 4A, Additional file 2 and Tables 1 and 2).

**Table 1:** Representative percentages of HCT11 p53^+/+ ^cells in the different cell cycle phases following Nutlin-3 or Etoposide treatment as described in Additional file 2.

p53+/+	subG1	G1	S	G2/M
Control	4.8	46.8	16.2	32.2
Nut 10 nM	3.1	59.1	11.9	25.9
Nut 15 nM	3.1	63.7	7.9	25.3
Nut 20 nM	3.1	66.0	5.9	25.0
Etoposide	20.9	17.1	12.0	50.0

**Table 2:** Representative percentages of HCT11 p53^-/- ^cells in the different cell cycle phases following Nutlin-3 or Etoposide treatment as described in Additional file 2.

p53-/-	subG1	G1	S	G2/M
Control	3.2	56.3	14.8	25.7
Nut 10 nM	5.7	19.5	25.3	49.5
Nut 15 nM	7.7	18.6	22.7	51.0
Nut 20 nM	7.2	18.3	22.7	51.8
Etoposide	10.2	13.7	19.1	57.0

### Nutlin-3 induces H2AX phosphorylation and foci formation

**Figure 5:**
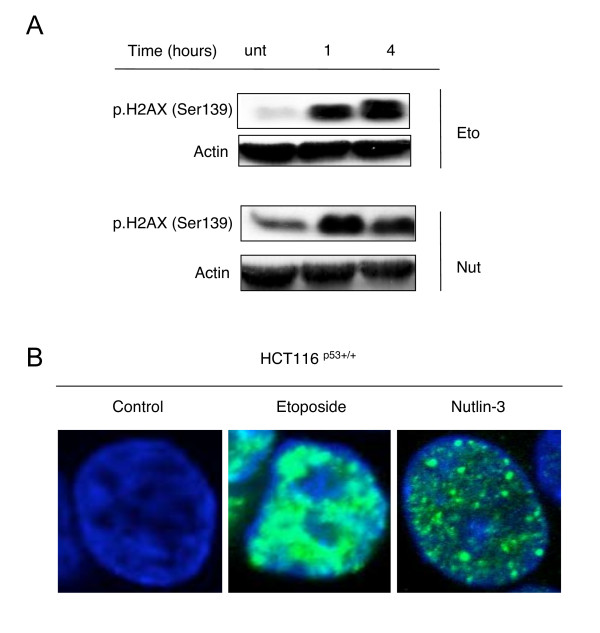
**Nutlin-3 induces H2AX phosphorylation and γH2AX foci in HCT116^p53+/+ ^cells**. *(A) *HCT116^p53+/+ ^cells were left untreated (unt) or treated with 100 μM Etoposide (Eto), 10 μM Nutlin-3 or (Nut) for 1 or 4 hours before the phosphorylation of H2AX (Ser139) was assessed using immunoblotting. Actin levels were used to assess equal loading. *(B) *Representative confocal microscopy images of γH2AX foci formation in HCT116^p53+/+ ^cells treated with either 100 μM Etoposide or 10 μM Nutlin-3 for 30 minutes.

One of the first proteins phosphorylated and activated in response to DNA-damage is the histone variant, H2AX [24]. Hence, we sought to investigate whether the observed Nutlin-3-dependent activation of ATM and CHK2 was due to a Nutlin-3-mediated DDR. Therefore, HCT116^p53+/+ ^cells were treated with either Etoposide or Nutlin-3, and H2AX phosphorylation was checked both 1 and 4 hours following treatment. Indeed, H2AX phosphorylation was induced in response to both Etoposide and Nutlin-3 treatment (Figure 5A).

We next sought to establish whether the observed Nutlin-3-induced activation of H2AX phosphorylation was indicative of γH2AX foci formation, an event recognised to occur early on in the DDR [24]. Indeed, treatment of HCT116^p53+/+ ^cells with Etoposide or Nutlin-3 was observed to induce γH2AX foci formation from as early as 30 minutes following treatment. Foci formation was most notable in response to Etoposide treatment, but was nevertheless clearly visible in response to treatment with Nutlin-3 (Figure 5B).

### Nutlin-3 induced responses are independent of p53 and Nutlin-3- mediated inhibition of MDM2

**Figure 6:**
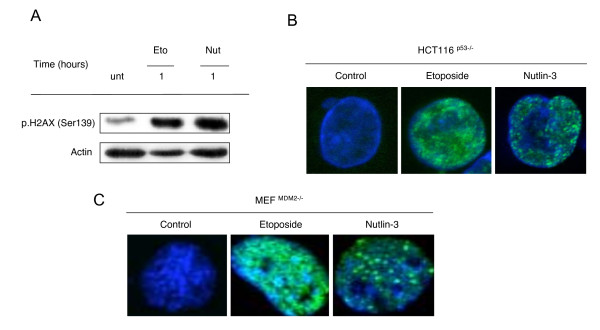
**Nutlin-3 induces H2AX phosphorylation and _H2AX foci formation independent of both p53 and MDM2 status**. *(A*) HCT116^p53-/- ^cells were left untreated (unt) or treated with 100 μM Etoposide (Eto) or 10 μM Nutlin-3 (Nut) for 1 hour before the phosphorylation of H2AX (Ser139) was assessed using immunoblotting. Actin levels were used to assess equal loading. *(B) *Representative confocal microscopy images of γH2AX foci formation in HCT116^p53-/- ^cells treated with either 100 μM Etoposide or 10 μM Nutlin-3 for 30 minutes. *(C) *Representative confocal microscopy images of γH2AX foci formation in MEF^MDM2-/- ^cells treated with either 100 μM Etoposide or 10 μM Nutlin-3 for 30 minutes.

We next sought to clarify the effect of p53 status on the ability of Nutlin-3 to induce the DDR. We therefore treated HCT116^p53-/- ^cells with Etoposide or Nutlin-3 and assessed the phosphorylation of γH2AX. Here, increases in γH2AX phosphorylation were observed in HCT116^p53-/- ^cells treated with either Etoposide or Nutlin-3 (Figure 6A). Furthermore, formation of γH2AX foci were clearly visible in HCT116^p53-/- ^cells following 30 minutes treatment with Etoposide, an effect which was comparable in cells treated with Nutlin-3 for the same time period (Figure 6B).

Having established that Nutlin-3 was capable of inducing DDR independent of p53 status, we went on to assess whether the ability of Nutlin-3 to induce DDR was dependent on its ability to inhibit MDM2. Here we assessed the effect of Nutlin-3 on formation of γH2AX foci in mouse embryonic fibroblasts deficient in MDM2 (MEF^MDM2-/-^). We observed clear formation of γH2AX foci after 30 minutes Nutlin-3 treatment, similar to that induced in cells treated with Etoposide for the same length of time (Figure 6C). Furthermore, in MEF^MDM2-/- ^cells, phosphorylation of ATM, ChK2, BRCA1 and γH2AX was noted following a 1 hour treatment with Nutlin-3, and markedly decreased by 24 hours (Additional file 3).

## Discussion

Numerous serine and threonine residues (mainly those located in the N-terminal part of the0020p53 protein) are targets for phosphorylation in response to a diverse range of stress factors. Following DNA-damage for instance, various protein kinases including ATM and CHK2 are activated and lead to p53 phosphorylation, subsequently resulting in stabilisation and activation of p53 [5-7].

The requirement of these phosphorylation events for the stabilisation and activation of p53 remains a somewhat controversial topic, as do the consequences of controlling the p53 pathway using the relatively newly developed MDM2 antagonists such as Nutlin-3. For example, there is debate as to whether MDM2 antagonism may affect p53 protein modifications or functions. A study carried out by Thompson *et al *using Nutlin-3, showed that phosphorylation of p53 on key serine residues was not necessary to bring about its stabilisation and activation. Indeed, whilst Thompson *et al *still observed stabilisation and activation of p53, no phosphorylation was detected following Nutlin-3 treatment [22].

In stark contrast, Drakos *et al *have since shown Nutlin-3-dependent induction of p53 phosphorylation at Ser15 in SP-53, Z-138, M-1 and Granta-519 MCL cell lines [23]. Nutlin-3-dependent p53 phosphorylation at Ser15 has also been observed in normal CD19^+ ^B-cells, peripheral blood mononuclear cells (PBMCs), bone marrow mononuclear cells (BMMCs) and B-CLL cells to a level similar to that noted in response to fludarabine treatment, and in excess of that resulting from treatment with the protease inhibitor clasto-latacystin [25]. Indeed in the current study, we observed Nutlin-3-induced stabilisation and activation of p53 at levels comparable with that induced by the genotoxic DNA topoisomerase II inhibitor; Etoposide (Figure 2A and 2B). We also detected Nutlin-3-induced phosphorylation of p53 at Ser15, as well as at two other key serine residues; Ser20 and Ser37 (Figure 3A), indicating that Nutlin-3 does not only disrupt the interaction between MDM2 and p53, but could also play a role in activating DDR pathways resulting in p53 phosphorylation, and subsequent activation of downstream target proteins involved in for example, cell cycle checkpoint control. Our results are in sharp contrast to the previous observations of Thompson *et al *[22]. In the current study, we checked p53 phosphorylation at earlier time points following Nutlin-3 treatment (as early as 2 hours, see Figure 2), however data in the Thompson *et al *study were obtained after 24 hour treatments with Nutlin-3, which could explain why such a difference is seen between the two studies. Indeed we also observed a marked decrease in these phosphorylations at 24 hours in response to Nutlin-3 (Additional file 1).

Since the activation of ATM and its downstream substrate CHK2 are well established as being responsible for DNA-damage-dependent p53 phosphorylation [5-7], we went on to investigate whether the observed Nutlin-3-dependent p53 phosphorylation was as a result of activation of these two kinases. Indeed, to our knowledge, we show for the first time that Nutlin-3 treatment triggers phosphorylation of ATM (Ser1981) and CHK2 (Thr68) in HCT116^p53+/+ ^cells (Figure 3B), demonstrating that Nutlin-3-mediated p53 phosphorylation is due to Nutlin-3 behaving as an activator of ATM and CHK2. Indeed our observation that Nutlin-3 also led to phosphorylation of a well established ATM target; BRCA1 (Ser1524) further supports a role for Nutlin-3 as an activator of the ATM kinase. Moreover, the phosphorylation of ATM and its target protein BRCA1 in HCT116^p53-/- ^cells (Figure 3C) suggests that the Nutlin-3-mediated activation of ATM and the subsequent phosphorylation of BRCA1 are triggered independently of p53.

Following DNA-damage, it is known that cells activate checkpoints to temporarily halt the cell cycle [26], allowing for DNA repair or destruction of the damaged cell by apoptosis. The G1-S and intra-S-phase checkpoints regulate transition into, and progression through S phase in response to DNA-damage, while the G2-M checkpoint regulates entry into mitosis [26]. Since ATM and CHK2 are amongst the main activators of these checkpoints in response to DNA-damage, we sought to determine whether cell cycle checkpoints could be triggered by Nutlin-3 treatment. Whilst Etoposide led to clear G2/M arrest, Nutlin-3 treatment led to marked G1/S arrest in HCT116^p53+/+ ^cells (Figure 4A), in keeping with the established role of p53 in triggering and maintaining G1/S arrest [27].

Conversely, in HCT116^p53-/- ^cells, Nutlin-3 led to G2/M arrest (Figure 4B), demonstrating Nutlin-3-mediated p53-independent induction of the G2/M cell cycle checkpoint, similar to that observed following Etoposide treatment. In addition, an increase in the sub-G1 cell population was also observed. Since sub-G1 is indicative of apoptotic cells, this suggests that Nutlin-3 may trigger p53-independent apoptosis. Given the absence of functional p53 in this instance, this prompted us to question whether Nutlin-3 was inducing the DDR without directly generating DNA-damage, or if the DDR was being activated due to Nutlin-3-induced DNA-damage.

One widely established indicator of DNA damage is the rapid phosphorylation of the histone variant H2AX at its C-terminal serine residue (Ser139) to form γH2AX, activation of which leads to its recruitment and subsequent accumulation (along with various repair proteins) into foci at the site of DNA damage [24]. Here, Nutlin-3 clearly induced the phosphorylation of H2AX (Figure 5A), and in addition was observed using immunofluorescent staining to cause clear γH2AX foci formation, similar to that observed in Etoposide-treated cells (Figure 5B). These findings demonstrate that Nutlin-3-dependent phosphorylation of p53 is due to the ability of Nutlin-3 to induce DNA-damage, or to otherwise activate pathways that are stimulated in response to DNA damage.

Recently, Verma *et al *have observed phosphorylation of H2AX in HCT116^p53+/+ ^following Nutlin-3 treatment. Nevertheless, an absence of γH2AX staining was noted by Verma *et al *unless Nutlin-3 was combined with treatment with the DNA damage inducer Hydroxyurea, and no phosphorylation of Ser15 was seen [28]. It is noteworthy that Verma *et al *observed these effects following a 24 hour treatment with Nutlin-3, whilst in the current study earlier time points were used after considering previous findings indicating that H2AX foci formation occurs as early as 1 minute after DNA-damage and peak at around 30-60 minutes [29-31], and previous observations that DNA-damage-induced stabilization and phosphorylation of p53 peak at 4-6 hours, declining thereafter [32,33].

Verma and colleagues attribute the induction of γH2AX staining to Nutlin-3-induced p53-mediated slowing of non-homologous end joining events following formation of DSBs during normal replicative processes, possibly as a way to ensure the accuracy of the repair process. However, in the current study we show Nutlin-3-induced phosphorylation of H2AX and formation of γH2AX foci in HCT116^p53-/- ^cells (Figure 6A and 6B). Coupled with the G2/M arrest we observed in p53 negative HCT116 cells, our data indicate that p53 is dispensable in the Nutlin-3-induced DDR. Furthermore, our observation that Nutlin-3 induces formation of γH2AX foci as well as ATM, ChK2 and BRCA1 phosphorylation in cells devoid of MDM2 (Figure 6C and Additional file 3), suggests that the secondary ability of Nutlin-3 to induce DNA-damage is not related to its primary function as an MDM2 antagonist.

## Conclusions

Direct inhibition of MDM2 using Nutlin-3 clearly provides a means of activating p53, and restoring p53 signaling, however in light of recent findings including those presented in the current study, we suggest Nutlin-3 is itself capable of instigating DNA-damage signaling. To our knowledge, we show for the first time that Nutlin-3 induces DDR activation in a p53-and MDM2-independent fashion. Further investigation is required to fully elucidate the effect of Nutlin-3 on p53-dependent and-independent DDR mechanisms, as well as its effects on the post-translational modification and functionality of p53, understanding of which will undoubtedly facilitate the development of Nutlin-3 and other MDM2 antagonists as potential cancer therapies.

## Competing interests

The authors declare that they have no competing interests.

## Authors' contributions

AM conceived of the study, whilst AM and JV were responsible for its design. JV carried out all assays relating to the study, including western blots, FACs and fluorescence microscopy. SK carried out some western blots. AM and JV analysed the data and drafted the manuscript. All authors read and approved the final manuscript.

## Pre-publication history

The pre-publication history for this paper can be accessed here:

http://www.biomedcentral.com/1471-2407/11/79/prepub

## Supplementary Material

Additional file 1 **Nutlin-3 leads to phosphorylation of key p53 Serine residues associated with DNA-damage**. HCT116^p53+/+ ^cells were untreated (treated with DMSO only) (unt) or treated with 100 μM Etoposide (Eto) or 10 μM Nutlin-3 (Nut) for the times indicated before immunoblotting was used to analyse phosphorylation of p53 at Ser15, Ser20 and Ser37. Actin levels were used to assess equal loading. Click here for file

Additional file 2 **Nutlin-3 induces p53-independent cell cycle checkpoint controls**. Representative histograms of HCT116^p53+/+ ^and HCT116^p53-/- ^cells following Nutlin-3 or Etoposide treatment. HCT116^p53+/+ ^and HCT116^p53-/- ^cells were treated with either 0, 10, 15 or 20 μM Nutlin-3 (Nut) or 100 μM of Etoposide (Eto). After 18 hours, cell cycle distribution was assessed using flow cytometry. Click here for file

Additional file 3 **Nutlin-3 leads to phosphorylation of several important DDR mediators, and results in phosphorylation of H2AX in MDM2 minus cells**. MEF^MDM2-/- ^cells were untreated (treated with DMSO only) (unt) or treated with 10 μM Nutlin-3 (Nut) or 100 μM of Etoposide (Eto) for 1 or 24 hours before the phosphorylation of ATM (Ser1981), BRCA1 (Ser1542), CHK2 (Thr68) and H2AX (Ser139) were analysed using immunoblotting. Actin levels were used to assess equal loading. Click here for file
