# Supplementary figures and images for: A p53-independent role for the MDM2 antagonist Nutlin-3 in DNA damage response initiation
Source: BMC Cancer. 2011 Feb 21;11:79. doi: 10.1186/1471-2407-11-79 (PMC3050855; doi:10.1186/1471-2407-11-79)

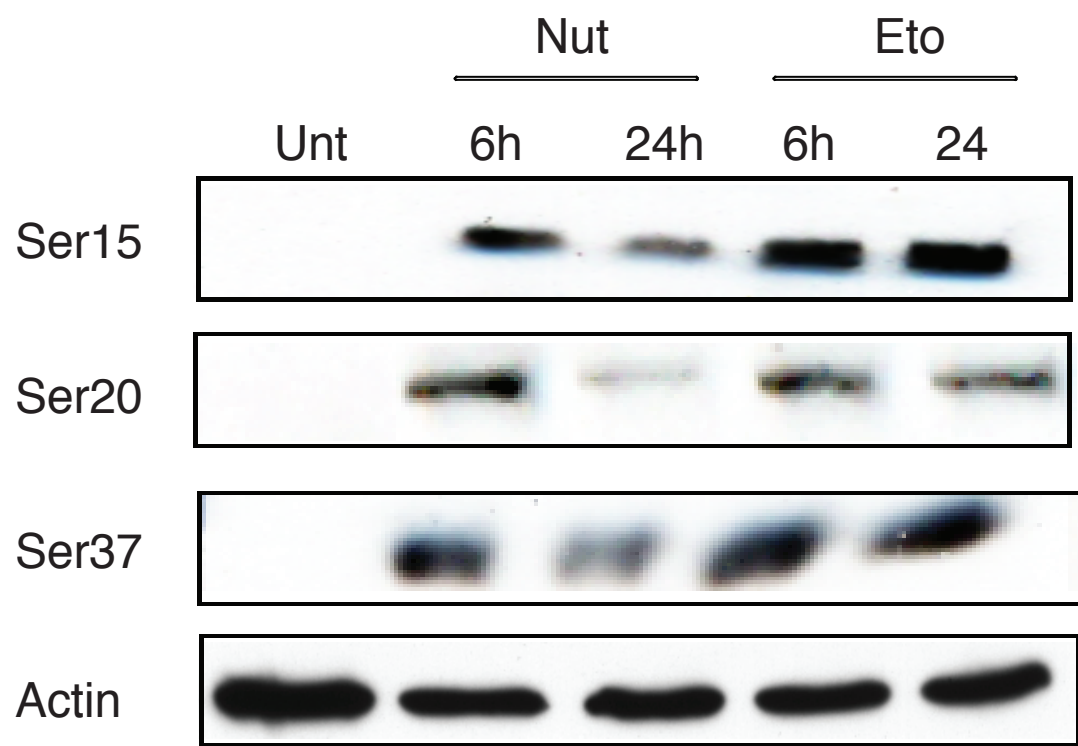

Supplement: Additional file 1 — Nutlin-3 leads to phosphorylation of key p53 Serine residues associated with DNA-damage. HCT116p53+/+ cells were untreated (treated with DMSO only) (unt) or treated with 100 μM Etoposide (Eto) or 10 μM Nutlin-3 (Nut) for the times indicated before immunoblotting was used to analyse phosphorylation of p53 at Ser15, Ser20 and Ser37. Actin levels were used to assess equal loading. [file 1471-2407-11-79-S1.PDF]

HCT116 p53<sup>+/+</sup>

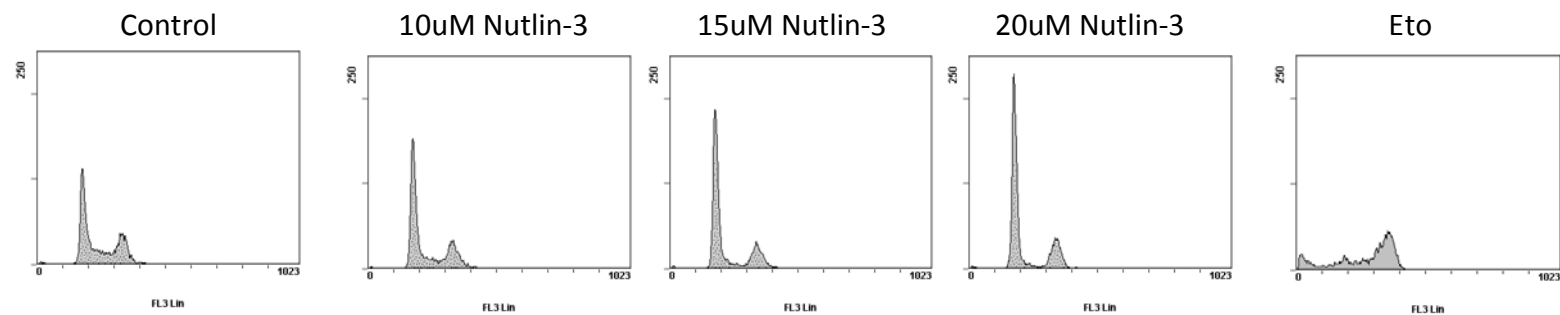

HCT116 p53<sup>-/-</sup>

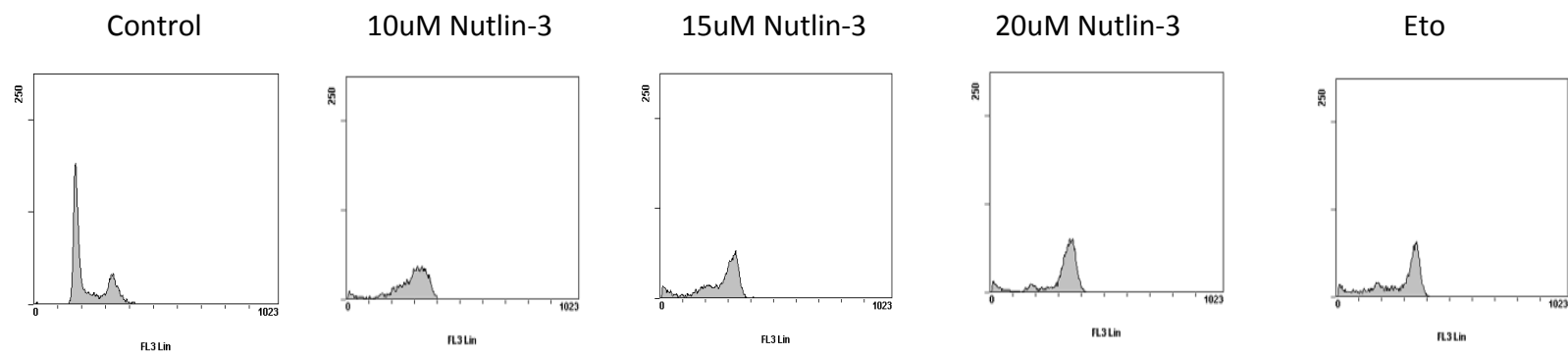

Supplement: Additional file 2 — Nutlin-3 induces p53-independent cell cycle checkpoint controls. Representative histograms of HCT116p53+/+ and HCT116p53-/- cells following Nutlin-3 or Etoposide treatment. HCT116p53+/+ and HCT116p53-/- cells were treated with either 0, 10, 15 or 20 μM Nutlin-3 (Nut) or 100 μM of Etoposide (Eto). After 18 hours, cell cycle distribution was assessed using flow cytometry. [file 1471-2407-11-79-S2.PDF]

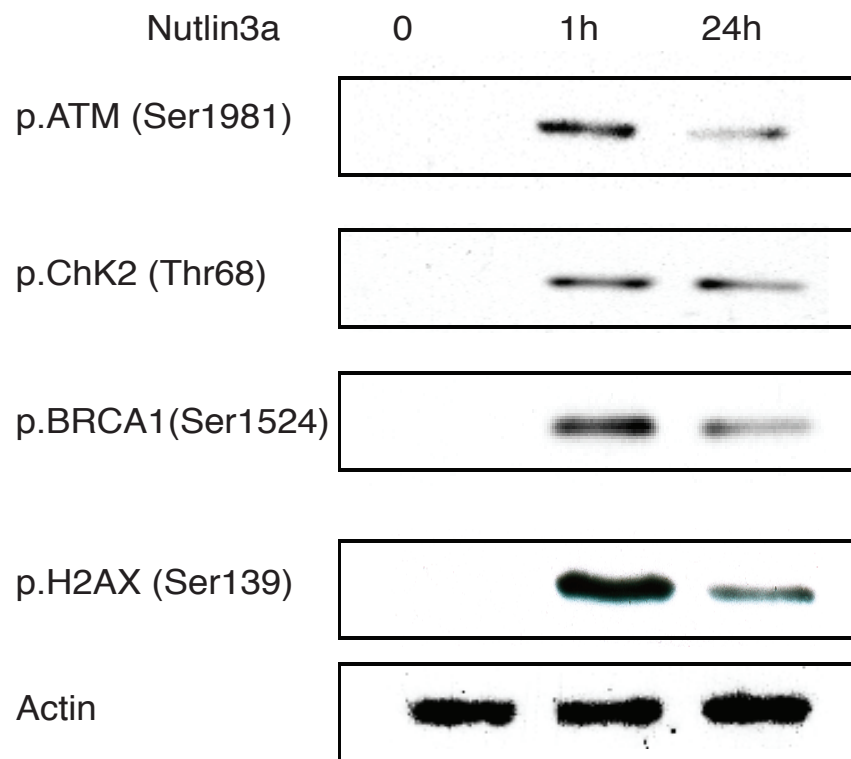

Supplement: Additional file 3 — Nutlin-3 leads to phosphorylation of several important DDR mediators, and results in phosphorylation of H2AX in MDM2 minus cells. MEFMDM2-/- cells were untreated (treated with DMSO only) (unt) or treated with 10 μM Nutlin-3 (Nut) or 100 μM of Etoposide (Eto) for 1 or 24 hours before the phosphorylation of ATM (Ser1981), BRCA1 (Ser1542), CHK2 (Thr68) and H2AX (Ser139) were analysed using immunoblotting. Actin levels were used to assess equal loading. [file 1471-2407-11-79-S3.PDF]
